# Association of gross domestic product with equitable access to childhood vaccines in 195 countries: a systematic review and meta-analysis

**DOI:** 10.1136/bmjgh-2024-015693

**Published:** 2025-01-19

**Authors:** Jerome Nyhalah Dinga, Jones Soladoye Akinbobola, Funmilayo Ibitayo Deborah Afolayan, Andreas Ateke Njoh, Tesfaye Kassa, David Dazhia Lazarus, Yakhya Dieye, Gezahegne Mamo Kassa, Kwabena Obeng Duedu, Nefefe Tshifhiwa, Mustapha Oumouna, AfVANET -African Vaccinology Network

**Affiliations:** 1 Michael Gahnyam Gbeugvat Foundation, Buea, Cameroon; 2 African Vaccinology Network, Buea, Cameroon; 3 University of Buea, Buea, Cameroon; 4 University of Abuja, Abuja, Nigeria; 5 University of Ibadan, Ibadan, Nigeria; 6 Expanded Program on Immunization, Ministry of Public Health, Yaounde, Cameroon; 7 School of Global Health and Bioethics, Euclid University, Bangui, Central African Republic; 8 Jimma University, Jimma, Ethiopia; 9 Food and Agriculture Organization of the United Nations, Abuja, Nigeria; 10 University Cheikh Anta Diop and Pasteur Institute, Dakar, Senegal; 11 Addis Ababa University, Addis Ababa, Ethiopia; 12 Birmingham City University, Birmingham, UK; 13 Biomedical Sciences, University of Health and Allied Sciences, Ho, Volta Region, Ghana; 14 ARC Onderstepoort Veterinary Research Campus, Onderstepoort, South Africa; 15 University of Médéa, Médéa, Algeria

**Keywords:** Vaccines, COVID-19, Global Health, Health policy, Immunisation

## Abstract

**ABSTRACT:**

**Introduction:**

Gross domestic product (GDP) has been shown to affect government spending on various budget heads including healthcare and the purchase and distribution of vaccines. This vulnerable situation has been exacerbated by the COVID-19 pandemic which disrupted and exposed the fragile nature of equitable access to vaccines for childhood immunisation globally. A systematic review and meta-analysis to assess the association of country income status and GDP with vaccination coverage of vaccines for childhood immunisation and other major infectious diseases around the globe will inform global and national policy on equity in living standards and vaccine uptake. This study was carried out to identify factors influenced by GDP that affect access, distribution, and uptake of childhood vaccines around the world using a systematic review and meta-analysis approach.

**Methods:**

Data were extracted for the burden of major infectious diseases of childhood immunisation programmes, factors affecting access to vaccines, vaccine procurement platforms, vaccination coverage and percentage of GDP used for the procurement of vaccines. Factors influencing the global vaccination coverage rate were also assessed. The protocol was registered on PROSPERO (ID: CRD42022350418) and carried out using Preferred Reporting Items for Systematic Reviews and Meta-Analyses guidelines.

**Results:**

Data from 195 countries showed that the following infectious diseases had the highest burden; human papillomavirus (HPV), measles, Ebola and yellow fever. Low-income and some lower-middle-income countries (LMICs) used COVAX and UNICEF for vaccine procurement while high-income countries (HICs) preferred national and regional public tenders. Global vaccination coverage for tuberculosis, diphtheria/tetanus/pertussis, hepatitis B, *Haemophilus influenzae* type b, measles, polio, meningitis and HPV had a significantly higher coverage than COVID-19. Being an HIC and having coverage data collected from 1985 to 2015 as the most current data were associated with high vaccination coverage. The percentage of GDP spent on vaccine procurement did not influence vaccination coverage.

**Conclusion:**

Low-income countries and LMICs should prioritise vaccine research and improve on development capacity. Countries worldwide should share data on vaccine expenditure, vaccination coverage, and the development and introduction of new vaccines and technologies to facilitate equitable vaccine access.

**WHAT IS ALREADY KNOWN ON THIS TOPIC**

- Infectious diseases targeted by childhood immunisation are the leading causes of death.
- COVID-19 has disrupted delivery of immunisation programmes.
- Gross domestic product (GDP) is associated with COVID-19 vaccine accessibility and uptake.
- Vaccination programmes remain highly vulnerable to budget cuts.
- Lack of equitable access to vaccines will hamper the eradication of vaccine-preventable diseases.

**WHAT THIS STUDY ADDS**

- Equitable access and vaccination coverage of childhood vaccines is associated with GDP in 195 countries.
- Data reported across World Bank income levels and disaggregated by the amount of GDP spent on vaccine procurement, vaccine procurement platform, and the year of availability of current vaccination coverage data.
- Data reported on disease incidence, death-to-case ratio and disability-adjusted life years to illustrate the true disease burden.

**HOW THIS STUDY MIGHT AFFECT RESEARCH, PRACTICE OR POLICY**

- Less developed countries should prioritise the enhancement of vaccine R&D capacity and the development and introduction of new vaccines and technologies.
- Developed countries should publish information on vaccination coverage and expenditure and share new vaccine technologies.
- Vaccine procurement platforms should do more to ensure global and national equitable access to vaccines.

## Introduction

 Infectious diseases that are targeted by childhood immunisation programmes are a subcomponent of vaccine-preventable infectious diseases that are leading causes of death, disease and disabilities worldwide.^1^ An estimated 700 000 children below the age of 5 years died of vaccine-preventable infectious diseases as of 2018^2^ with 99% occurring in low-income and middle-income countries. Childhood immunisation has been demonstrated to be a cost-effective tool to eliminate and eradicate infectious diseases of poverty and vaccine-preventable infectious diseases. It remains an important aspect of primary and preventive healthcare and is directly responsible for saving millions of lives.^3^,^5^

While the COVID-19 pandemic has shown that the use of vaccines is essential to protect the health and well-being of the population, it has also exposed and significantly worsened inequitable access to vaccines between countries.^6^,^9^ These enhanced vaccine inequalities are strongly felt in routine immunisation coverage with inequalities within a country,^10 11^ between countries^12^ and in different regions of the world.^13^,^15^

Sustainable financing is one of many integral elements that contribute to successful vaccination programmes which require consistent resources and long-term commitment.^16^ However, vaccination programmes remain highly vulnerable to budget cuts with tiny fractions of the gross domestic product (GDP) being spent on vaccines. In a recent survey, 4 (Ghana, Malawi, Tanzania and Zimbabwe) out of 33 African countries did not have any line items related to immunisation.^17^

Research on different vaccines in various countries has shown that immunisation uptake is related to the same factors associated with other health inequities and multifactorial social determinants of health, for example, age, education and household income level.^18^,^24^ The cost of a vaccination programme in the USA can be increased by the fact that some regions receive more vaccines than are needed while fewer are supplied to others.^25^ In order to forecast demand for influenza vaccine, one project estimated that there will be a rise of 7.7% in demand^26^ but this type of forecast does not give specific information to guide those managing supply chains or manufacturers to produce enough vaccines to prepare for a pandemic or influenza season. The logistic and transport system of a country affects its ability to promptly deliver acquired vaccines to where they are needed.^27^ In low/lower-middle income countries (LMICs) where the physical supply chain, from cold chain considerations to transport capacity, is already stressed, it is clear that vaccine delivery and uptake will be varied in these circumstances.^28^ Mis/disinformation and lack of trust in the health system could fuel vaccine hesitancy and hamper vaccine uptake strategies.^19^,^2329^

The availability of data has hampered the monitoring and surveillance of the coverage and effectiveness in some countries.^30^ Bureaucratic hurdles and lack of political will can limit the availability of vaccination coverage data.^31^ Wrong estimates of the population can lead to inaccurate estimates of zero-dose and underimmunised children.^32^ Other factors that can hamper the availability of vaccination coverage data include; legal and ethical barriers, lack of resources and personnel, selection and recall bias, survey implementation, data quality, and reporting.^1533^,^36^

It has been proposed that national, regional and global stakeholders should come together and seek ways to achieve justice with countries having equitable access to vaccines by enhancing vaccine development capacity,^37 38^ sharing of technology and regional manufacturing. State actors, manufacturers and donors could develop strategies for financing and political will to ensure predictable demand, equitable access to vaccines, and vaccination at national and subnational levels for all countries.^38 39^

If there is no equitable access to vaccines for infectious diseases, these diseases will continue to persist for a much longer time leading to the loss of many lives and liberty. In addition to saving lives and averting illnesses, expanding equitable access to good quality vaccines between countries also helps to advance economic development.^40 41^ Previous studies have shown that GDP is associated with COVID-19 vaccination coverage^1942^,^45^ and in low-income countries (LICs)^46^ but no association has been assessed between GDP and equitable childhood vaccines access and vaccination coverage globally.

In this systematic review and meta-analysis, we looked at the association of GDP with equitable access and vaccination coverage for vaccines used in childhood immunisation programmes around the world and compared it to COVID-19 vaccination coverage in 195 countries. We also aimed at identifying factors influenced by GDP that affect access, distribution and uptake of childhood vaccines around the world.

## Methods

### Study design

We conducted a cross-sectional quantitative analysis of existing data to explore the trends, progress and equity of vaccine distribution and vaccination coverage for infectious diseases targeted in childhood immunisation programmes and COVID-19, globally. We evaluated the contribution of various procurement mechanisms, income level, percentage of GDP spent on vaccine procurement and year of data availability using publicly available information and databases.

Two researchers independently screened the records for inclusion in this study. Researchers were blinded to each other’s decisions. A third researcher resolved any disagreement between the two scientists screening the records for inclusion in the study.

### Data sources

A search for online databases was conducted between 05 September 2023 and 13 January 2024. Key information sources for the quantitative analysis included; Google Scholar, PubMed Central, WHO, Africa Centres for Disease Control and Prevention (Africa CDC), Worldometer, Our World in Data (OWID), Gavi, the Vaccine Alliance, Save the Children, blogs, online reports, social listening, research theses and the UNICEF Vaccine Market Dashboard.

Epidemiological, geographical and demographic data were extracted from these information sources. This included World Bank (WB) income classification level; LICs, LMICs, upper-middle income countries (UMICs) and high-income countries (HICs),^47^ and the burden of diseases targeted in childhood immunisation programmes and COVID-19. Death-to-case ratios were obtained directly from databases^48^ or calculated from the following; number of deaths per year divided by number of cases per year.

For data on variables such as vaccination coverage, year of coverage and vaccine procurement details, we extracted information from published articles (cross-sectional, longitudinal, reviews and systematic reviews), blogs, grey literature and manuscripts, the UNICEF Vaccine Market Dashboard database, OWID and WHO, simultaneously incorporating data from the WB for categorisation of countries based on income level.

Percentage of GDP spent on vaccine procurement for each country was obtained directly from internet searches. For countries whose percentage GDPs spent on vaccine procurement were not publicly available, this was calculated from the following; GDP, expenditures on vaccines per capita, percentage health budget for vaccines, percentage of GDP for health budget^49^ and the country’s population.^50^

Search terms included: GDP per country, expenditures on vaccines per capita, percentage health budget for vaccines, percentage of GDP for health budget, country population, childhood vaccination, approved COVID-19 vaccines, vaccines for vaccine-preventable infectious diseases, WHO prequalified vaccines, infectious childhood diseases, vaccine procurement platforms, vaccination coverage.

### Vaccination coverage

Vaccination coverage was obtained from WHO and UNICEF^51^ that contains information and data that are varying and, in some instances, unknown.^51 52^ For diseases with more than one vaccine or dose,^51 53 54^ the average coverage was calculated^55^ and reported for that disease and year per country.

### Inclusion criteria

- The title of the literature is consistent with the objectives of study.
- The abstract, when present, is consistent with the objectives of the study.
- Literature on equitable vaccine access for vaccine-preventable infectious diseases.
- Only studies in English.
- No time frame was used to reduce the possibility of losing studies and perform a comprehensive analysis.
- The most recent data available online for each country was used.
- Income category for each country was used as classified by the WB.

### Exclusion criteria

- The title of the literature is not consistent with the objectives of the study.
- The abstract, when present, is not consistent with the objectives of the study.
- Literature on topics other than equitable vaccine access for vaccine-preventable infectious diseases.
- Literature in languages other than English.

### Risk of bias assessment

**Figure 1:**
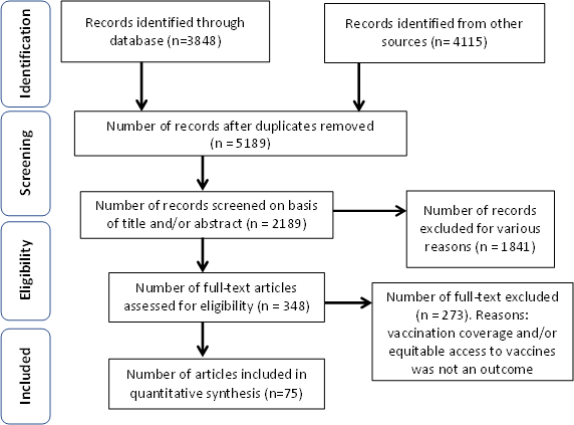
Preferred Reporting Items for Systematic Reviews and Meta-Analyses (PRISMA) flow diagram of the literature search. From a total of 5189 studies identified (following both the initial and updated literature review in the select databases), we screened 2189 studies for eligibility (using the search term ‘childhood vaccination’), removed 1841 duplicates, and excluded 273 studies not reporting vaccination coverage and/or equitable access to vaccines as an outcome. Therefore, we finally included a total of 75 eligible studies/reports that mentioned vaccination coverage as an outcome for this systematic review and meta-analysis.

There have been controversies surrounding the best practices of risk of bias assessment.^56^ Even though other studies have recommended against past practices focusing on reporting quality, relying solely on study design or numerical quality scores, and automatically downgrading for industry sponsorship to select studies for systematic reviews of healthcare interventions,^56^ risk of bias assessment for records included in this systematic review and meta-analysis was done using the Agency for Healthcare Research and Quality checklist for Risk of Bias assessment, which included checking for: defining the source of information, indicating time, proper characterisation of subjects, assessment for quality assurance, explaining how data were handled, summarising completeness of data. Each item scores one point. Based on the scores, records were grouped into three; articles into three: low, moderate and high. Records with moderate and high scores were synthesised. Figure 1 is a flow chart of how the records were selected for this systematic review and meta-analysis. Preferred Reporting Items for Systematic Reviews and Meta-Analyses guideline as implemented in this study is shown in online supplemental material S1.

### Secondary data analysis

Countries were divided into HICs, UMICs, LMICs and LICs based on the WB income group categorisation,^47^ and used for secondary quantitative and regression analysis. The most recent data available online was used for each country and income group as classified by the WB.^47^

For multivariable regression analysis, percentage GDP was categorised into low (0%–0.0098% GDP on vaccine procurement), medium (0.00981%–0.019% GDP on vaccine procurement) and high (0.02%–0.5% GDP on vaccine procurement) expenditure. Vaccination coverage was ranked into low (0%–50% vaccination coverage), medium (50.1%–80% vaccination coverage) and high (80.1%–100% vaccination coverage) coverage, while coverage year was categorised into the following ranges; 1985–2015, 2016–2020 and 2021–2023. These categories are all arbitrary as there is no citable literature that clearly categorises them. WHO, CDC and UNICEF mention vaccination coverage as percentages but have not categorised coverage per se.

### Patient and public partnership

This research did not involve any patient or the general public at large. Only online information was used in the present study.

### Statistical analysis

Data were collected in Microsoft Excel and imported into R programming (R V.4.2.1) for analysis and visualisation.^57^ Two-sample t-test performed between each of the income levels to identify one that had a significantly higher coverage. Two-way analysis of variance was performed to determine if WB income level was associated with high vaccination coverage. Pearson’s product-moment correlation coefficient was used to determine the correlation between percentage GDP spent on vaccine procurement and vaccination coverage. Logistic regression was used to identify factors associated with high vaccination coverage.

### Protocol registration

The protocol for this analysis has been registered at www.prospero https://www.crd.york.ac.uk/prospero/ under ID: CRD42022350418.

## Results

### Demographic analysis

Seventy-five studies involving 195 countries were included in this study; 62 HIC, 53 UMIC, 54 LMIC and 26 LIC. The analysis presents the global burden of diseases targeted in childhood immunisation programmes, factors affecting equitable access to vaccines for childhood immunisation, financing of vaccines, vaccine procurement platforms and vaccination coverage across countries in the world.

### Burden of major vaccine-preventable infectious diseases

A comparative and consistent description of the burden of diseases and their risk factors is an important input to health decision-making and planning processes. The disability-adjusted life year (DALY) is a single measure to quantify the burden of diseases. The DALY is based on years of life lost from premature death and years of life lived in less than full health. ‘One DALY can be thought of as one lost year of ‘healthy’ life and the measured disease burden is the gap between a population’s health status and that of a normative reference population’.^48^ DALYs for a specific cause are calculated as the sum of the years of life lost from mortality from that cause and the years of healthy life lost due to disability (YLDs) for people living in states of less than good health resulting from the specific cause. The average global relative uncertainty (%) for YLD by cause shows that yellow fever has the highest burden of disease (online supplemental table S1). Based on DALY and available data, yellow fever had the highest disease burden (online supplemental table S1).

### Percentage of GDP and the actual amount used for vaccine procurement

**Figure 2:**
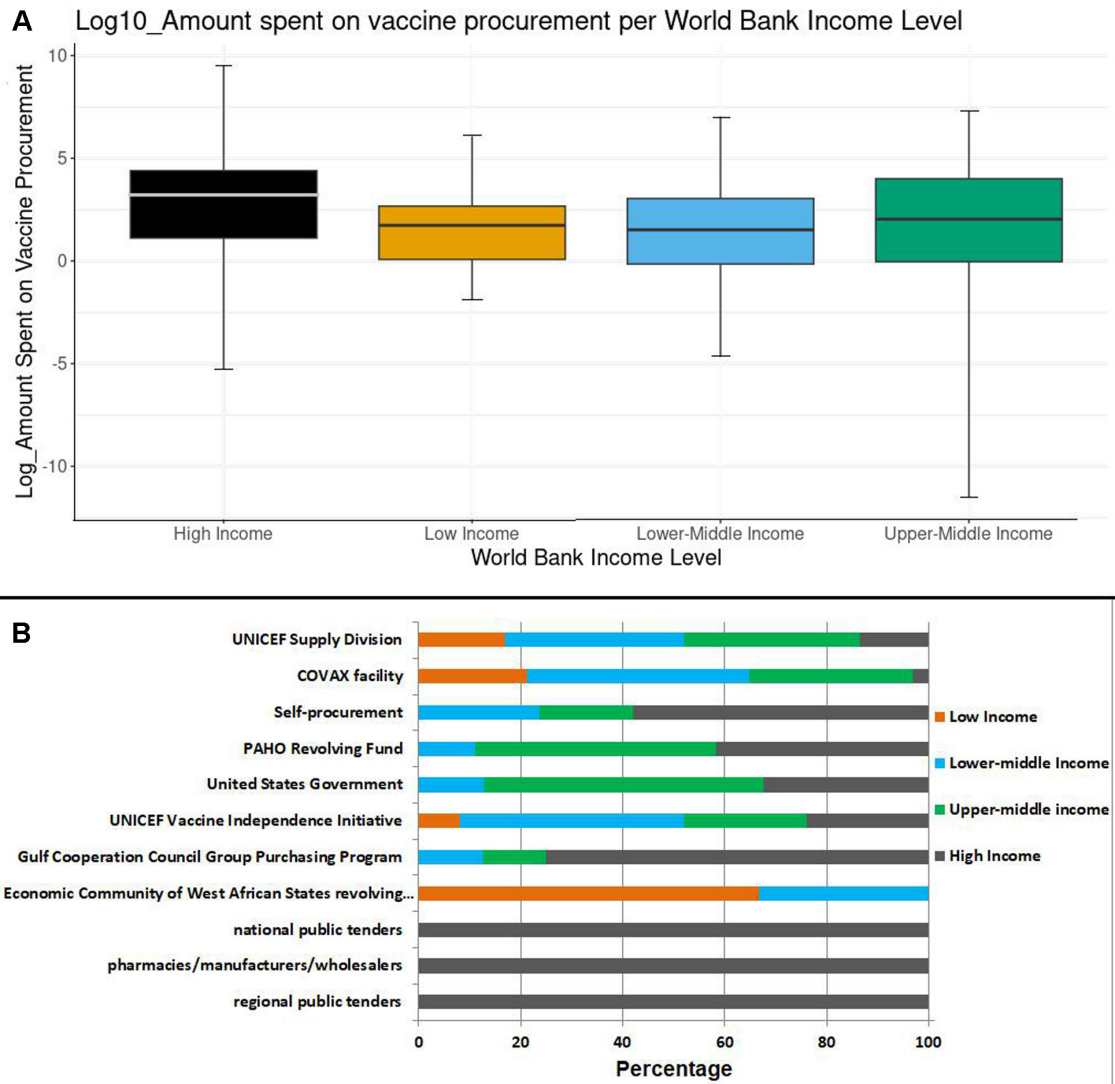
Vaccine procurement. (A) Log_10_ of the amount of gross domestic product (GDP) spent on vaccine procurement and grouped according to the World Bank income level classification. (B) Major vaccine procurement platforms used in 195 countries around the world. PAHO, Pan American Health Organization.

There was a significant difference in the percentage of GDP each country used to procure vaccines across the various income levels (p<0.04). When the per cent GDP was converted to the actual amounts in million US$, the difference among the different WB income levels was even greater (p<0.008). Figure 2 shows the log_10_ of the actual amounts spent on vaccine procurement across the different WB income level categorisations.

### Vaccine procurement platforms

Of the countries using the UNICEF Supply Division, 86.5% (78) of them were LICs, LMICs or UMICs while these income levels made up 96.8% (91) of the countries using the COVID-19 Vaccines Access (COVAX) facility (figure 2B).

There was a statistically significant difference in the manner in which countries of different income levels use the different platforms for vaccine procurement (p<0·001) (figure 2B). WB income level was associated with the vaccine procurement platform used by LICs having preference for the UNICEF Supply Division (p<0.002), COVAX facility (p<0.009), UNICEF Vaccine Independence Initiative (UNICEF VII) (p<0.0007), and Economic Community of West African States revolving fund (p<0.009). While HICs had preference for self-procurement (p<0.02), Pan American Health Organization Revolving Fund (p<0.0001), US government (p<0.0001), Gulf Cooperation Council Group Purchasing Program (p<0.0001), national public tenders (p<0.0001), pharmacies/manufacturers/wholesalers (p<0.0001) and regional public tenders (p<0.0001) procurement platforms (figure 2B).

### Vaccination coverage rate in relation to GDP

This analysis covers vaccines for major infectious diseases targeted during childhood immunisation programmes and other major infectious diseases. These include vaccines against tuberculosis (TB vaccines), diphtheria/tetanus/pertussis (DTP vaccines), neonatal tetanus (Protected at birth (PAB) vaccine), hepatitis B (HepB vaccines), *Haemophilus influenzae* type b (Hib vaccines), measles (measles vaccines), polio (polio vaccines), gravid tetanus (gravid tetanus vaccines), meningitis (meningitis vaccines), rotavirus (rotavirus vaccines), yellow fever (yellow fever vaccines), rubella (rubella virus), human papillomavirus (HPV vaccines) and COVID-19 (COVID-19 vaccines).

**Figure 3:**
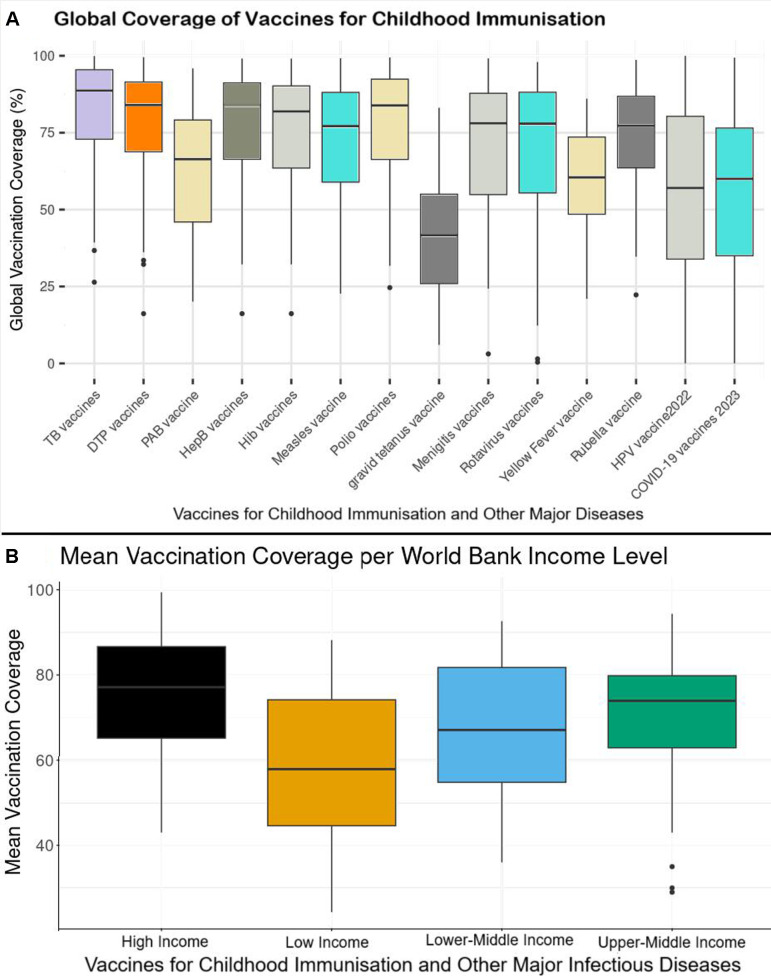
Vaccination coverage rate in relation to GDP. (A) Global vaccination coverage for common vaccine-preventable diseases. (B) Vaccination coverage per World Bank income level. DTP, diphtheria/tetanus/pertussis; GDP, gross domestic product; HepB, hepatitis B; Hib, *Haemophilus influenzae* type b; HPV, human papillomavirus; TB, tuberculosis.

Latest and most recent available vaccination coverage data were used ranging from 1985 for some countries and up to 2023 for others. As of 2023, TB was the disease with the highest global vaccination coverage of 83.09% while gravid tetanus had the least vaccination coverage of 40.92% (figure 3A). As of March 2023, COVID-19 vaccination (full dose) had a global coverage rate of 53.16% (figure 3A).

The following vaccines had a better coverage than COVID-19; TB vaccines (p<0.002), DTP vaccines (p<0.000), HepB vaccines (p<0.000), Hib vaccines (p<0.001), measles vaccines (p<0.000), polio vaccines (p<0.000), meningitis vaccines (p<0.018), and HPV vaccines (p<0.014) but not PAB vaccines (p<0.683), gravid tetanus vaccines (p<0.606), rotavirus vaccines (p<0.713), yellow fever vaccines (p<0.408), and rubella vaccines (p<0.147) (figure 3A).

**Figure 4:**
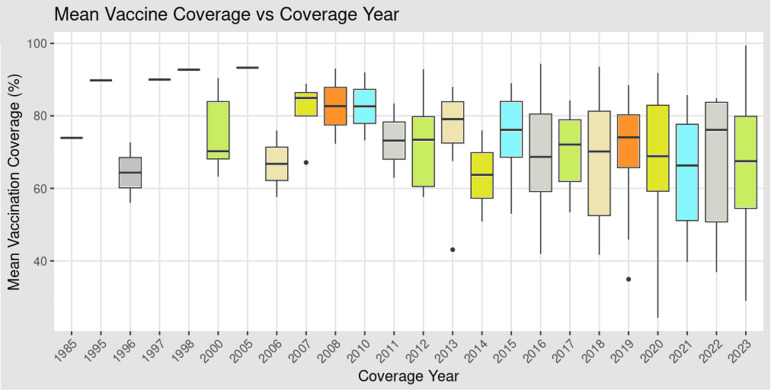
Average vaccination coverage across the globe and associated factors. Mean vaccination coverage rates were plotted against the coverage year for which the most current data were available.

It was observed that LICs had the lowest overall mean vaccination coverage rate compared with the other income levels; LICs (59.26%), LMICs (67.87%), UMICs (70.27%) and HICs (75.65%) (p<0.0001) (figure 4).

Compared with LICs, LMICs (p<0.018), UMICs (p<0.003) and HICs (p<0.0001) had a significantly higher mean vaccination coverage (figure 3B).

The vaccination coverage for the following vaccines was significantly different across the WB income levels; TB vaccines (p<0.0001), HepB vaccines (p<0.0001), Hib vaccines (p<0.0003), measles vaccines (p<0.0000), polio vaccines (p<0.0000), meningitis vaccines (p<0.03655) and COVID-19 vaccines (p<0.0000).

It was observed that there was a significant negative correlation between vaccine coverage year and percentage GDP (p<0.002) with data available in the earlier years having a higher vaccination coverage rate as compared with data available in the latter years (figure 4). There was no correlation between mean vaccination coverage rates and percentage of GDP spent on vaccine procurement across the WB income levels (p<0.8665). It was also observed that LICs and LMICs had more recent data compared with UMICs and HICs.

### Factors associated with high vaccination coverage rate and equitable access to vaccines

It is important to combine and measure multivariate inequity over multiple groups to access factors associated with vaccination coverage. A few determinants were identified as being associated with high vaccination coverage for vaccine-preventable diseases.

The determinants investigated were: year of availability of coverage data, WB income level and percentage of GDP spent on vaccine procurement. Not every country had their coverage data updated to the year 2023 online, so the latest data for each country that were available online were used and were referred to as ‘year of availability of coverage data’ for that country.

**Figure 5:**
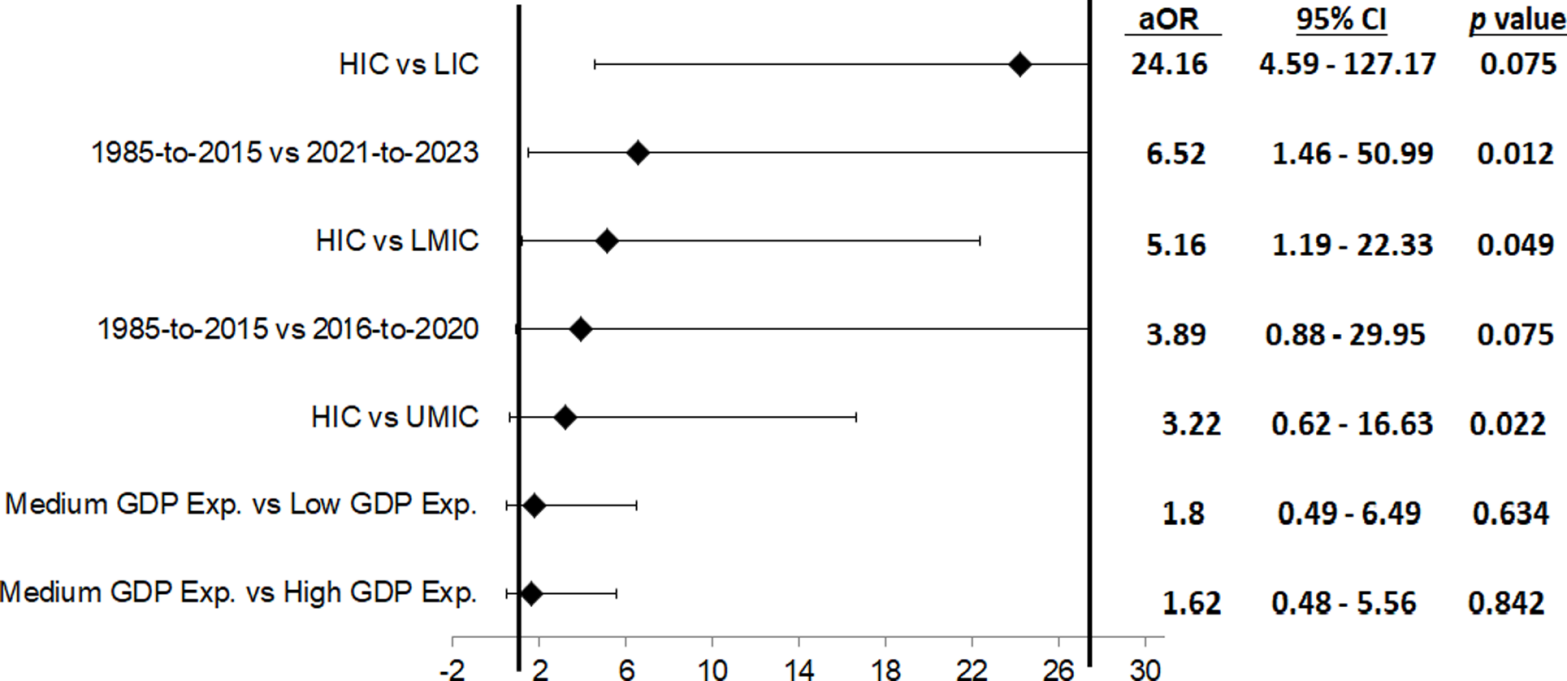
OR analysis to identify determinants of high vaccination coverage and to account for potential confounding variables to global vaccination coverage in relation to old vaccination coverage rates online (1985–2015) and medium expenditure of GDP on vaccine procurement. The following variables were adjusted for: LIC, LMIC, UMIC, low GDP expenditure on vaccine procurement, high GDP expenditure on vaccine procurement, vaccination coverage from 2016 to 2023. Percentage GDP was categorised into low (0%–0.0098% GDP on vaccine procurement), medium (0.00981%–0.019% GDP on vaccine procurement), and high (0.02%–0.5% GDP on vaccine procurement) expenditure. Vaccination coverage was ranked into low (0%–50% vaccination coverage), medium (50.1%–80% vaccination coverage) and high (80.1%–100% vaccination coverage) coverage. aOR, adjusted OR; GDP, gross domestic product; HIC, high-income country; LIC, low-income country; LMIC, low-middle-income country; UMIC, upper-middle-income country.

The odds that countries have high vaccination coverage rates when their coverage data were from 1985–2015 is 3.89 times the odds that countries whose coverage data were from 2016–2020 to have high coverage rates. Also, the odds that countries have high vaccination coverage when their coverage data were from 1985–2015 is 6·52 times the odds that countries whose coverage data were from 2021–2023 to have high coverage rates. In other words, the odds that countries have high coverage rates is increased by 289% when available coverage data are from 1985–2015 compared with data from 2016–2020, and by 552% compared with available data from 2021–2023. However, this difference is only statistically significant for comparison done between data from 1985–2015 and 2021–2023 (p<0.012) but not for the comparison of coverage data from 1985–2015 and 2016–2020 (p<0.075) (figure 5).

HICs had a higher odds of obtaining high vaccination coverage compared with LMICs (OR=5.15, 95% CI 1.19 to 22.33, p<0.049), UMICs (OR=3.22, 95% CI 0.62 to 16.63, p<0.022) and LICs (OR=24.16, 95% CI 4·59 to 127·17, p<0.000) (figure 5).

When the level of expenditure (percentage GDP) on vaccine procurement was assessed as a determinant of equitable access to vaccines and vaccination coverage, it was observed that the percentage GDP spent on vaccine procurement did not have any impact on vaccination coverage (figure 4).

## Discussion

In this study we aim to establish the association between GDP and access to vaccines for childhood immunisation and other major diseases. This was also carried to identify factors that influence vaccination coverage in 195 countries. Ensuring vaccine security in all countries around the world is one way to guarantee the prevention, control and elimination of major infectious diseases targeted by childhood immunisation. Even though HICs had a high vaccination coverage rate, it was also observed that countries with similar GDP levels had different vaccination coverage rates, which could be due to economic inequalities, political stability and external aid programmes.^4558^,^61^ It is well understood that we need multitasked and multilinked complex activities to prevent or control epidemics, endemics and pandemics.^62^,^71^ This implies that there is no one-size-fits-all policy mix any country can adopt but rather follow a contextualised approach for a common health goal for the general benefit of all.^4072^,^75^ This study attempted to identify and elaborate on some of the factors that could influence a public health outcome like childhood immunisation coverage for vaccine for routine vaccination as well as vaccines for other major infectious diseases. We also looked at GDPs associated with these factors.

Using the death-to-case ratios and DALYs, this study identifies HPV, measles, Ebola and yellow fever as the most deadly vaccine-preventable infectious diseases, whereas another study identified TB as the vaccine-preventable infectious disease with the highest disease burden.^76^

Vaccination has contributed enormously to global health with two major infections, smallpox and rinderpest, having been successfully eradicated. Global coverage of vaccination against many major infectious diseases targeted during childhood immunisation programmes has been enhanced significantly since the creation of the Global Alliance for Vaccination and Immunization (Gavi, the Vaccine Alliance) in 2000. As compared with a WHO study carried out in 2022,^77^ this study shows an increase in global coverage for HPV, rotavirus, rubella and yellow fever. But there was no difference in the global coverage for Hib, meningitis, measles, and maternal and neonatal tetanus while a decreased coverage for HepB, DTP and polio was observed in this study compared with another study.^77^

One of the major setbacks to equitable access to vaccines is the mechanisms and procedures involved in the procurement of vaccines around the world. Vaccine procurement should be implemented as a continuous development, with each process directly as well as indirectly informing the next. The global effect of COVID-19 has implied huge changes in the way administrative associations work to procure vaccines for vaccine-preventable infectious diseases.^78^ Our study showed that while LICs and LMICs relied on international networks to procure vaccines, HICs tended to buy directly from manufacturers or through national or regional tenders. This tendency is expected as the economies of poor countries have been depleted over the last 2 years due to the COVID-19 pandemic. Hence, the opportunities presented by the UNICEF Supply Division, COVAX facility and UNICEF VII provided the much-needed lifeline for LICs, LMICs and some UMICs to acquire vaccines for vaccine-preventable diseases.^79^ UNICEF VII enables governments to manage temporary budget shortfalls and facilitate timely procurement of vaccines among other supplies. As shown by our study and other studies,^80^ HICs have the tendency to use national public tenders, regional public tenders, direct purchase of vaccines by general practitioners, and pharmacies for vaccine procurement.

It was observed that there was a significant difference in the percentage GDP spent on vaccine procurement between the different WB income levels.^19^ This was contrary to an earlier study which showed that there was no statistically significant difference between the different WB income levels, as far as the percentage GDP spent on vaccine procurement is concerned. This is probably due to the fact that more countries were studied in this meta-analysis compared with the previous study.^19^

With the many factors that affect the manufacture, purchase and distribution of vaccines, it is important that accurate and up-to-date data be made available by all countries to guide the international community to understand the intricacies of vaccine equity and shed light on the blind spots essential for achieving global equitable access to vaccines for vaccine-preventable diseases.^81^ Unfortunately, the present quantitative synthesis indicates that HICs do not share up-to-date data on vaccination coverage and expenditure on vaccine procurement.

Previous studies assessing the association of GDP with vaccination coverage have either focused on COVID-19^42^,^45^ or LICs^46^ but none has looked at the percentage GDP spent on vaccine procurement and its association to vaccination coverage of childhood vaccines (an indicator of equitable access to vaccines). This systematic review and meta-analysis is the first study to look at the percentage GDP spent on vaccines and its association with vaccination coverage and equitable access to childhood vaccines in 195 countries.

The strength of the study is that it analysed global vaccination coverage and expenditure on vaccines from 195 countries. Country-specific up-to-date expenditure on vaccine procurement was also included and factors associated with global and national equitable access to vaccines from the perspective of GDP were identified.

One weakness of the study is the lack of public data on up-to-date vaccination coverage and expenditure on vaccine procurement for all countries and some had to be calculated using publicly available supporting data. The years for which the most recent vaccination coverage data that were available online for some countries were different, thereby limiting the analysis as countries were compared against each other for data that were collected in different years. Another limitation was that not all vaccines used in the Global North are available in the Global South. Only records in English were included because of the lack of funding and time for professional translators. The estimates of incidence, mortality and DALY were extracted from varying years.

## Conclusion

Being a HIC, having medium expenditure on vaccine procurement and not releasing current data on vaccination coverage online strongly increased the chances of having high vaccination coverage of vaccines for childhood immunisation and other major infectious diseases.

Boosting efficiency in the use of government funds will be critical in ensuring equitable access to vaccines globally. One way of doing so is to empower regional^37^ and national capacity to ensure local vaccine development and manufacturing.^82^ This will ensure reduction in vaccine prices, preferential purchasing arrangements and delivery costs. However, it should be noted that other factors like global cooperation,^83^,^86^ funding mechanisms^8187^,^89^ and political stability^690^,^92^ could influence the effectiveness of the aforementioned strategies.

## supplementary material

10.1136/bmjgh-2024-015693 online supplemental file 1

10.1136/bmjgh-2024-015693 online supplemental table 1

## Data Availability

Data are available upon reasonable request.
