# Supplementary material for: Association of gross domestic product with equitable access to childhood vaccines in 195 countries: a systematic review and meta-analysis
Source: BMJ Glob Health. 2025 Jan 19;10(1):e015693. doi: 10.1136/bmjgh-2024-015693 (PMC11749592; doi:10.1136/bmjgh-2024-015693)
Supplement: online supplemental file 1 [file bmjgh-10-1-s001.pdf]

PRISMA 2020 Checklist

Section and Topic	Item #	Checklist item	Location where item is reported
**TITLE**			
Title	1	Association of Gross Domestic Product with Equitable Access to Childhood Vaccines in 195 Countries: a systematic review and meta-analysis	Page 1
**ABSTRACT**			
Abstract	2	**Introduction**<br>Gross Domestic product has been to affect government spending on various budget heads include health and purchase and distribution of vaccines. This vulnerable situation has been exacerbated by COVID-19 pandemic has disrupted and exposed the fragile nature of equitable access to vaccines for childhood immunization globally. A systematic review and meta-analysis to assess the association of country income status and gross domestic product with vaccination coverage of vaccines for childhood immunization and other major infectious diseases around the globe will inform global and national policy on equity on living standards and vaccine uptake. This study was carried out to identify factors influenced by gross domestic product that affect access, distribution and uptake of childhood vaccines around the world using a systematic review and meta-analysis approach.<br><br>**Methods**<br>Data was extracted for the burden of major infectious diseases of childhood immunization programs, factors affecting access to vaccines, vaccine procurement platforms, vaccination coverage, and percentage of gross domestic product used for the procurement of vaccines. Factors influencing the global vaccination coverage rate were also assessed. The protocol was registered on PROSPERO (ID: CRD42022350418) and carried out using PRIMSA guidelines.<br><br>**Results**<br>Data from 195 countries showed that the following infectious diseases with had the highest burden; Human papillomavirus, Measles, Ebola, and Yellow Fever. Vaccine price was identified as an important factor for equitable access to vaccines. Poor countries used COVAX and UNICEF for vaccine procurement while rich countries preferred national and regional public tenders. Global vaccination coverage for Tuberculosis, Diphtheria-Tetanus-Pertussis, Hepatitis B, *Haemophilus influenzae* type b, Measles, Polio, Meningitis, and Human papillomavirus had a significantly higher coverage than COVID-19. Being a high-income country and data collected from 1985 to 2015 were associated with high vaccination coverage. Percentage of Gross Domestic Product spent on vaccine procurement did not influence vaccination coverage.<br><br>**Conclusion**<br>Less developed countries should prioritize the development of vaccine research and development capacity. Countries worldwide should share data on vaccine expenditure, vaccination coverage, and the development and introduction of new vaccines and technologies to facilitate equitable vaccine access.	Page 1
**INTRODUCTION**			

PRISMA 2020 Checklist

Section and Topic	Item #	Checklist item	Location where item is reported
Rationale	3	A systematic review and meta-analysis to assess the association of country income status and gross domestic product with vaccination coverage of vaccines for childhood immunization and other major infectious diseases around the globe will inform global and national policy on equity on living standards and vaccine uptake.	Page 1, 2
Objectives	4	1. To use systematic review and meta-analysis to look at the association of GDP with equitable access and vaccination coverage for vaccines used in childhood immunization programs around the world and compared it to COVID-19 vaccination coverage in one hundred and ninety-five (195) countries.<br>2. To identify factors influenced by GDP that affect access, distribution and uptake of childhood vaccines around the world.	Page 1 & 3
**METHODS**			
Eligibility criteria	5	**Inclusion criteria**<br>• The title of the literature is consistent with objectives of study<br>• The abstract, when present, is consistent with objectives of the study<br>• Literature on equitable vaccine access for vaccine preventable infectious diseases<br>• Only studies in English<br>• No time frame used in order to reduce the possibility of losing studies and perform a comprehensive analysis.<br>• The most recent data available online for each country was used.<br>• Income category for each country was used as classified by the WB.<br><br>**Exclusion Criteria**<br>• The title of the literature is not consistent with objectives of study<br>• The abstract, when present, is not consistent with objectives of the study<br>• Literature on topics other than equitable vaccine access for vaccine preventable infectious diseases<br>• Literature in languages other than English.	Page 4
Information sources	6	Specify all databases, registers, websites, organisations, reference lists and other sources searched or consulted to identify studies. Specify the date when each source was last searched or consulted.	
Search strategy	7	Present the full search strategies for all databases, registers and websites, including any filters and limits used.	
Selection process	8	Specify the methods used to decide whether a study met the inclusion criteria of the review, including how many reviewers screened each record and each report retrieved, whether they worked independently, and if applicable, details of automation tools used in the process.	
Data collection process	9	Specify the methods used to collect data from reports, including how many reviewers collected data from each report, whether they worked independently, any processes for obtaining or confirming data from study investigators, and if applicable, details of automation tools used in the process.	
Data items	10a	List and define all outcomes for which data were sought. Specify whether all results that were compatible with each outcome domain in each study were sought (e.g. for all measures, time points, analyses), and if not, the methods used to decide which results to collect.	

PRISMA 2020 Checklist

Section and Topic	Item #	Checklist item	Location where item is reported
	10b	List and define all other variables for which data were sought (e.g. participant and intervention characteristics, funding sources). Describe any assumptions made about any missing or unclear information.	
Study risk of bias assessment	11	There have been controversies surrounding the best practices of risk of bias assessment. Even though, other studies have recommend against past practices focusing on reporting quality, relying solely on study design or numerical quality scores, and automatically downgrading for industry sponsorship to select studies for systematic reviews of healthcare interventions, risk of Bias assessment for records included in this systematic review and meta-analysis was done using the Agency for Healthcare Research and Quality checklist for Risk of Bias assessment, which included checking for; defining the source of information, indicating time, proper characterization of subjects, assessment for quality assurance, explaining how data were handled, summarizing completeness of data.	Page 4
Effect measures	12	Not carried out	
Synthesis methods	13a	item #5.	Page 4
	13b	Data was collected into a spread sheet and imported into R for cleaning and further analysis	Results section
	13c	Data analysis and visualization was done in R Programming software	results
	13d	Data analysis and visualization was done in R Programming software	Results section
	13e	Data analysis and visualization was done in R Programming software	Results section
	13f	Sensitivity analyses not done. Packages in R Programming were used for their robustness and accuracy	Results section
Reporting bias assessment	14	Agency for Healthcare Research and Quality checklist for Risk of Bias assessment was used to check for; defining the source of information, indicating time, proper characterization of subjects, assessment for quality assurance, explaining how data were handled, summarizing completeness of data. Each item scores one point. Based on the scores, records were grouped into three; articles into three: low, moderate and high. Records with moderate and high score were synthesized.	Page 4
Certainty assessment	15	Each item scores one point. Based on the scores, records were grouped into three; articles into three: low, moderate and high. Records with moderate and high score were synthesized.	Page 4

PRISMA 2020 Checklist

Section and Topic	Item #	Checklist item	Location where item is reported
**RESULTS**			
Study selection	16a	Describe the results of the search and selection process, from the number of records identified in the search to the number of studies included in the review, ideally using a flow diagram.	

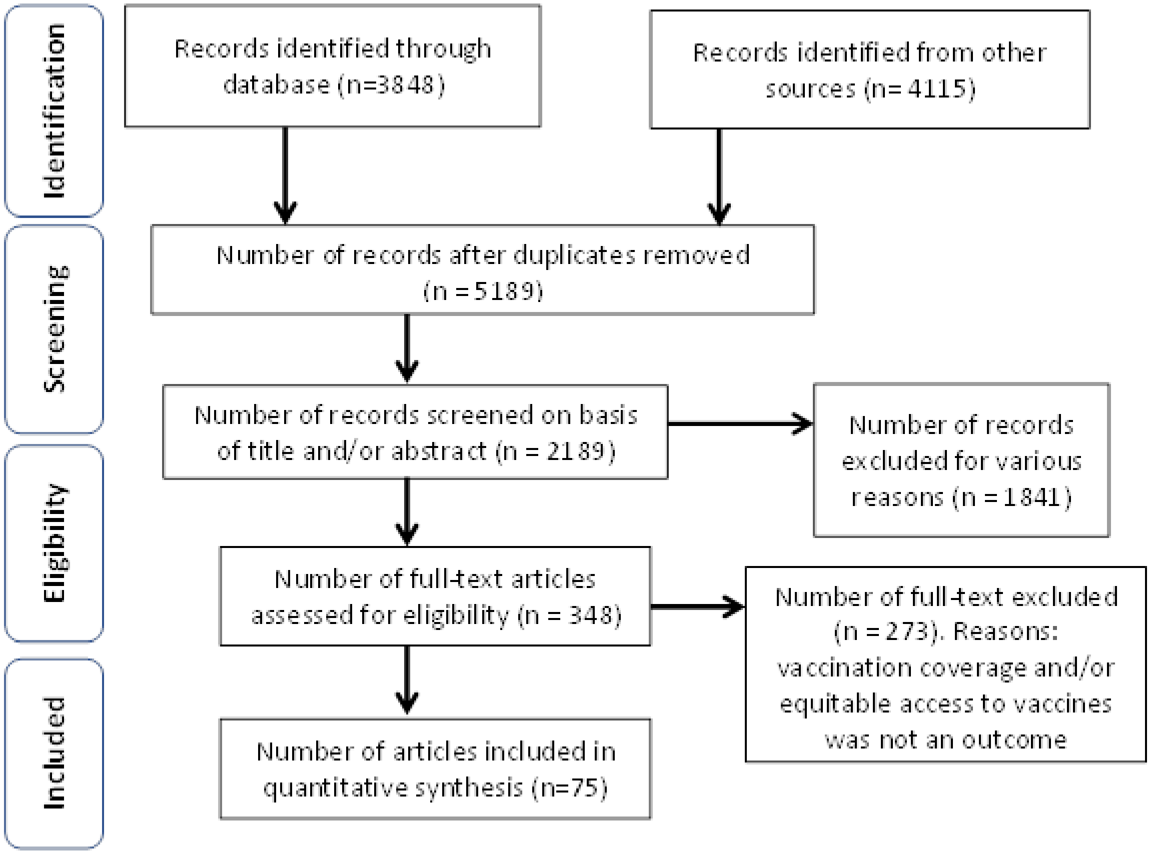

Section and Topic	Item #	Checklist item	Location where item is reported
	16b	All study/literature that met inclusion criteria were included.	Results section
Study characteristics	17	Cite each included study and present its characteristics.	Results section
Risk of bias in studies	18	risk of Bias assessment for records included in this systematic review and meta-analysis was done using the Agency for Healthcare Research and Quality checklist for Risk of Bias assessment, which included checking for; defining the source of information, indicating time, proper characterization of subjects, assessment for quality assurance, explaining how data were handled, summarizing completeness of data.	Page 4
Results of individual studies	19	effect estimate and its precision was not done for each individual literature was not calculated	Results sections
Results of syntheses	20a	For each synthesis, briefly summarise the characteristics and risk of bias among contributing studies.	
	20b	(see figure below)	
	20c		
	20d		
Reporting biases	21		
Certainty of evidence	22		

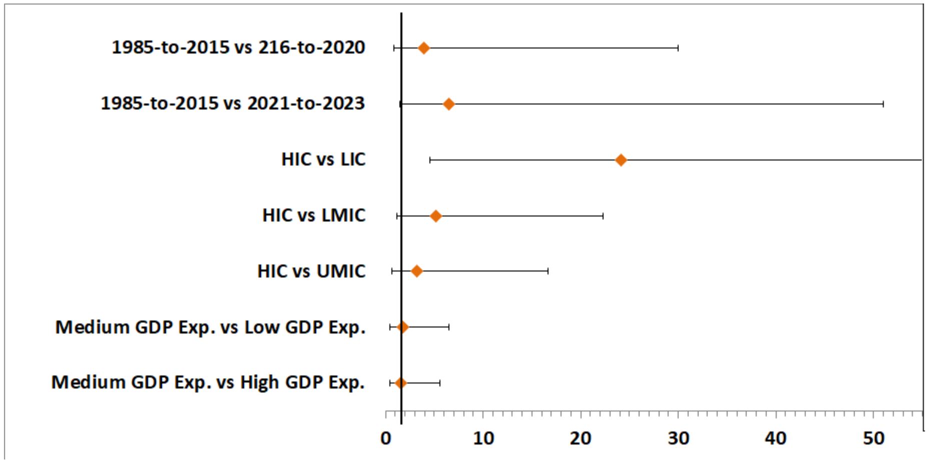

Odds Ratio	95% CI	*p* value
3.89	0.88 - 29.95	0.075
6.52	1.46 - 50.99	0.012
24.16	4.59 - 127.17	0.000
5.16	1.19 - 22.33	0.049
3.22	0.62 - 16.63	0.022
1.8	0.49 - 6.49	0.634
1.62	0.48 - 5.56	0.842

Multiple logistic regression analysis to identify determinants of high vaccination coverage.

Section and Topic	Item #	Checklist item	Location where item is reported
**DISCUSSION**			
Discussion	23a	• Equitable access and vaccination coverage of childhood vaccines is associated with Gross Domestic Product in 195 countries.<br>• Data reported across World Bank income levels and disaggregated by percentage gross domestic product spent on vaccine procurement, vaccine procurement platform, and the year of availability of the current vaccination coverage data.<br>• Data reported on disease incidence, death-to-case ratio and Disability-adjusted life years (DALYs) to illustrate the true disease burden.	Page 2, 16
	23b	One weakness of the study is the lack of public data on up-to-date vaccination coverage and expenditure on vaccine procurement for all	Page 17

PRISMA 2020 Checklist

Section and Topic	Item #	Checklist item	Location where item is reported
		countries and some had to be calculated using publicly available supporting data. Another is that, not all vaccines used in the global North are available in the global South.	
	23c	Only records in English were included because of lack of funding and time for professional translators. The estimates of incidence, mortality, DALY were extracted from varying years.	Page 17
	23d	• Less developed countries should prioritize enhancement of vaccine R&D capacity and the development and introduction of new vaccines and technologies.<br>• Developed countries should publish information on vaccination coverage and expenditure and share new vaccine technologies.<br>• Vaccine procurement platforms should do more to ensure global and national equitable vaccine access.	Page 2
**OTHER INFORMATION**			
Registration and protocol	24a	PROSPERO ID: CRD42022350418	Page 5
	24b	https://www.crd.york.ac.uk/prospero/ under ID: CRD42022350418	Page 5
	24c	The original protocol was written to cover only access to COVID-19 vaccine in Africa but later edited to include childhood vaccines in the world	Page 5
Support	25	No external funding was received for this study	
Competing interests	26	No competing interests was declared	
Availability of data, code and other materials	27	Raw data is available upon request from corresponding author with due diligence.	

From: Page MJ, McKenzie JE, Bossuyt PM, Boutron I, Hoffmann TC, Mulrow CD, et al. The PRISMA 2020 statement: an updated guideline for reporting systematic reviews. BMJ 2021;372:n71. doi: 10.1136/bmj.n71. This work is licensed under CC BY 4.0. To view a copy of this license, visit https://creativecommons.org/licenses/by/4.0/
