# Supplementary material for: Association of gross domestic product with equitable access to childhood vaccines in 195 countries: a systematic review and meta-analysis
Source: BMJ Glob Health. 2025 Jan 19;10(1):e015693. doi: 10.1136/bmjgh-2024-015693 (PMC11749592; doi:10.1136/bmjgh-2024-015693)
Supplement: online supplemental table 1 [file bmjgh-10-1-s002.pdf]

Supplementary Table S1: Worldwide incidence and death-to-case ratio of common vaccine preventable diseases and WHO recommended vaccines

	Disease	Cases/year	Death/year	Death-to-Case Ratio	Average global relative uncertainty (%) for YLD by cause	Major Available vaccines/WHO prequalified vaccines
1.	Anthrax	2,000	unavailable	unavailable	unavailable	BioThrax, Anthrax Vaccine Adsorbed
2.	Cholera	535,321	4,000	0.007	unavailable	Euvichol–S vaccine.
3.	COVID-19	88,678/1 million people [58]	885/1 million people [58]	0.009/1 million people	unavailable	Nuvaxovid, Spikevax, Comirnaty, Convidecia, Jcovden, Vaxzevria, Covishield, Bimervax, JN.1 monovalent vaccine, and KP.2 monovalent vaccines
4.	Dengue	7.6 million	3000	0.0004	125.5	Dengue vaccine, Dengvaxia
5.	Diphtheria	17,000	1,700	0.1	124.2	DTaP vaccine, Tdap vaccine, Td vaccine, Adsorbed DT Vaccine, Diftet, Diphtheria and Tetanus Vaccine Adsorbed (Paediatric), Diphtheria and Tetanus Vaccine Adsorbed for Adults and Adolescents, IMOVAX dT adult. Tetadif, Adacel, Boostrix, Hexaxim, Diphtheria-Tetanus-Pertussis Vaccine Adsorbed, DTP Vaccine, TRIPVAC, Diphtheria, Tetanus, Pertussis and Haemophilus influenzae type b Conjugate Vaccine, Diphtheria, Tetanus, Pertussis, Hepatitis B and Haemophilus

						influenzae type b Conjugate Vaccine, Easyfive-TT, Eupenta, ComBE Five, Pentabio
6.	Ebola	28,616	11,310	0.39	unavailable	Mvabea, Zabdeno, ERVEBO
7.	*Haemophilus influenzae* type b (Hib)	7 – 8 million	100,000	0.0125 – 0.0143	unavailable	DTaP-IPV/Hib vaccine, Hexaxim, Diphtheria, Tetanus, Pertussis and Haemophilus influenzae type b Conjugate Vaccine, Diphtheria, Tetanus, Pertussis, Hepatitis B and Haemophilus influenzae type b Conjugate Vaccine, Easyfive-TT, Eupenta, ComBE Five, Pentabio, Act-HIB, Haemophilus influenzae type b Conjugate Vaccine, Quimi-Hib
8.	Hepatitis A	1.4 million	7,000	0.005	101.8	Havrix, Vaqta, HEALIVE, Havrix 720 Junior
9.	Hepatitis B	30 – 260 million	900,000	0.003 – 0.03	84.1	Engerix, Euvax B, Heberbiovac HB, Hepatitis B Vaccine, Hexaxim, Diphtheria, Tetanus, Pertussis, Hepatitis B and Haemophilus influenzae type b Conjugate Vaccine, Easyfive-TT, Eupenta, ComBE Five, Pentabio
10.	Human papillomavirus	662,301	348,874	0.53	unavailable	Gardasil 9, Gardasil, Cervarix, Cecolin, Gardasil, Walrinvax,
11.	Influenza	1 billion	290,000 – 650,000	0.0003 – 0.0007	unavailable	Afluria Quadrivalent, Fluarix Quadrivalent, FluLaval Quadrivalent, Fluzone Quadrivalent, Celtura, Focetria, Green Flu-S, Influenza A (H1N1) 2009

						monovalent vaccine, NASOVAC Influenza Vaccine, Panenza, Panvax, Pandemic Live Attenuated Vaccine, GC FLU Quadrivalent inj., Influvac, SKYCellflu Quadrivalent inj., VaxigripTetra, GC FLU Multi inj., GC FLU inj, IL-YANG FLU Vaccine INJ., influenza trivalent vaccine (split virion, inactivated), Nasovac-S Influenza Vaccine, Live, Attenuated (Human), Serinflu, SKYCellflu® inj., SKYCellflu® Multi inj., Vaxigrip
12.	Japanese encephalitis	68,000	15,000 – 20,000	0.2 – 0.3	38	IXIARO, JEEV® (3µg), JEEV® (6µg), IMOJEV MD, Japanese Encephalitis Vaccine Live (SA14-14-2)
13.	Malaria	249 million	608,000	0.002	55.7	Mosquirix, CYVAC
14.	Measles	350,000	140,000	0.4	113.3	Measles vaccine, Measles Vaccine, Live, Attenuated, MMRV vaccine, Measles and Rubella Vaccine (Live) (Attenuated, Freeze Dried), Measles and Rubella Vaccine, Live, Attenuated, Priorix, rHA M-M-R II
15.	Meningitis	500,000	50,000	0.1	42.6	MenFive, Meningococcal A Conjugate MenAfriVac, Meningococcal A Conjugate 5 micrograms MenAfriVac 5µg, Menactra, Menveo, Nimenrix, MenQuadfi

16.	Mumps	500,000	unavailable	unavailable	unavailable	MMRV vaccine, Priorix, rHA M-M-R II
17.	Pertussis	24 million	161,000	0.007	55.3	DTaP vaccine, Tdap vaccine, Adacel, Boostrix, Hexaxim, Diphtheria-Tetanus-Pertussis Vaccine Adsorbed, DTP Vaccine, TRIPVAC, Diphtheria, Tetanus, Pertussis and Haemophilus influenzae type b Conjugate Vaccine, Diphtheria, Tetanus, Pertussis, Hepatitis B and Haemophilus influenzae type b Conjugate Vaccine, Easyfive-TT, Eupenta, ComBE Five, Pentabio
18.	Pneumococcal disease	unavailable	1,000,000	unavailable	unavailable	PCV, PNEUMOSIL, Prevenar 13, Prevenar 13 Multidose Vial, Synflorix, Prevnar 20, Pneumo23;
19.	Polio	35	unavailable	unavailable	unavailable	IPV Vaccine AJV, Picovax, Poliomyelitis vaccine, Poliomyelitis Vaccine (Inactivated), Poliomyelitis vaccine multidose, ShanIPV, Polio Vaccine - Novel Oral (nOPV) Type 2, Hexaxim, IMOVAX POLIO, Eupolio Inj., Poliomyelitis Vaccine (Vero Cell), Inactivated, Sabin Strains, BIOPOLIO B1/3, Bivalent OPV Type 1 and 3 Poliomyelitis Vaccine, Live (Oral), Bivalent Oral Poliomyelitis Vaccine Type 1&3 (bOPV 1&3), Polio Sabin One and Three,

						Poliomyelitis Vaccine (live, oral attenuated, human Diploid Cell), type 1 and 3, Poliomyelitis Vaccine (Oral), Bivalent types 1 and 3, Monovalent Oral Poliomyelitis Vaccine Type 1 (mOPV1), Polio Sabin Mono T1, Polio Sabin Mono Two (oral), ORAL MONOVALENT TYPE 2 POLIOMYELITIS VACCINE (mOPV2), Monovalent Oral Poliomyelitis Vaccine Type 2, Polio Sabin Mono Three (oral), BIOPOLIO, Oral Poliomyelitis Vaccines (Oral Drops), Polioviral vaccine
20.	Rabies	29 million	59,000	0.002	152.7	HDCV vaccine, PCEC vaccines, Rabies Vaccine Inactivated (Freeze Dried)(RABIVAX-S), VaxiRab N, VERORAB
21.	Respiratory Syncytial virus	3.3 million	101,400	0.03	unavailable	RSV vaccine, Arexvy, mResvia, Abrysvo
22.	Rotavirus	258 million	128,000	0.0005	41.1	RotaTeq, Rotarix, ROTASIIL, ROTASIIL-Liquid, ROTASIIL®Thermo, Rotavac, ROTAVAC 5D®
23.	Rubella	15,000	unavailable	unavailable	unavailable	MMRV vaccine, Measles and Rubella Vaccine (Live) (Attenuated, Freeze Dried), Measles and Rubella Vaccine, Live, Attenuated, Priorix, rHA M-M-R II, Rubella Vaccine, Live, Attenuated

24.	Tetanus	0.5 – 1 million	40,000	0.04 – 0.08	115.9	DTaP vaccine, Tdap vaccine, Td vaccine, Diphtheria and Tetanus Vaccine Adsorbed (Paediatric), Diphtheria and Tetanus Vaccine Adsorbed for Adults and Adolescents, IMOVAX dT adult, Tetadif, Adacel, Boostrix, Hexaxim, Diphtheria-Tetanus-Pertussis Vaccine Adsorbed, DTP Vaccine, TRIPVAC, Diphtheria, Tetanus, Pertussis and Haemophilus influenzae type b Conjugate Vaccine, Diphtheria, Tetanus, Pertussis, Hepatitis B and Haemophilus influenzae type b Conjugate Vaccine, Easyfive-TT, Eupenta, ComBE Five, Pentabio, BEtt, Tetanus Toxoid Vaccine Adsorbed, Tetatox, TETAVAX, TT vaccine
25.	Tuberculosis	10 million	1.5 million	0.15	74.5	bacille Calmette-Guerin
26.	Typhoid fever	11 – 21 million	128,000 – 161,000	0.008 – 0.012	unavailable	SKYTyphoid Multi Inj., Typbar-TCV, TYPHIBEV
27.	Varicella (Chickenpox)	40 – 50 million	4,200	0.00008 – 0.0001	unavailable	BARYCELA inj., SKYVaricella Inj., Varicella Vaccine, Live, Varivax.
28.	Yellow Fever	84,000 – 170,000	30,000 – 60,000	0.353 – 0.357	156.2	SinSaVac, Stabilized Yellow Fever Vaccine, STAMARIL , Yellow Fever, live-attenuated yellow fever vaccine 17D
